# Association between malocclusion and other occlusions in mixed and permanent dentitions: a cross-sectional study

**DOI:** 10.3389/froh.2026.1726950

**Published:** 2026-03-13

**Authors:** Sarah Ahmed Bahammam, Hammam Ahmed Bahammam

**Affiliations:** 1 Preventive Dental Sciences Department, Dental College and Hospital, Taibah University, Medina, Saudi Arabia; 2 Department of Paediatric Dentistry, Faculty of Dentistry, King Abdulaziz University, Jeddah, Saudi Arabia

**Keywords:** dental caries, gingival recession, malocclusion, mixed dentitions, oral health, tooth wear

## Abstract

**Introduction:**

Malocclusion, a dental abnormality commonly found in children, has been associated with a risk of developing other occlusion problems. In the literature, no comparison has been made as to whether children with mixed or permanent dentition are more predisposed to occlusion problems from malocclusion. The present study aims to investigate various occlusion problems that may arise later in life due to malocclusion.

**Methods:**

This cross-sectional study included 500 children below the age of 12 years at Taibah University Hospital (Aug 2024–April 2025). Convenience sampling was used to recruit participants, who were also excluded if they had undergone previous orthodontic treatment or had craniofacial abnormalities. Standardized intraoral clinical examinations were conducted to assess malocclusion, dentition type, tooth wear, traumatic dental injury, and gingival recession. The data were analyzed using SPSS-v22 with descriptive statistics and chi-square and logistic regression analyses to list factors related to malocclusion.

**Results:**

In analyses, malocclusion was associated with tooth wear and permanent dentition. In multivariable analysis, tooth wear (adjusted OR = 0.69; 95% CI: 0.49–0.99) and permanent dentition (adjusted OR = 0.64; 95% CI: 0.45–0.91) were inversely associated with malocclusion, while traumatic dental injury was not. Model explanatory power was low (Nagelkerke *R*^2^ = 0.028).

**Discussion:**

Tooth wear and permanent dentition were inversely associated with malocclusion, whereas traumatic dental injury and mixed dentition were not. The low explanatory power highlights the multifactorial and developmentally dynamic nature of malocclusion, suggesting that occlusal irregularities during dentition transition may be transient rather than predictive of persistent malocclusion.

## 1 Introduction

Malocclusion is defined as misaligned teeth or an abnormal relation between the two dental arches i.e., upper and lower (1). The prevalence of malocclusion is 56%, meaning that nearly one out of two individuals suffers from this dental abnormality (2). Malocclusion ranks as the third most significant oral health concern, after dental caries and periodontal diseases (3). The necessity and demand are also rising for orthodontic treatment due to the increased prevalence of dental diseases including malocclusions (4). It has a diverse aetiology wherein both environmental and genetic factors are influential (5).

The principal cause of most malocclusions are external factors such as tooth loss, compromised oral care, mouth tumours, thumb sucking, bottle feeding, and cleft lip and palate (6–10). It usually occurs in children during their mixed dentition stage (5), but can also be found in children irrespective of their age (11). Malocclusion can inflict a negative self-image and can adversely affect the oral health-related quality of life (OHRQoL) (12). In addition to psychological distress, this may lead to other occlusion problems later in life if not treated in a timely. Malocclusion can impair a person's ability to break down food hence reducing the masticatory performance (13). Large overjet might increase the chance of traumatic dental injury (TDI) by two or threefold to both anterior permanent and primary teeth. It accounts for approximately 100–300 million TDIs globally (14). Malocclusions such as maxillary incisor crowding and spacing, mandibular incisor crowding, and irregularity can lead to the progression of periodontitis (15), an inflammatory disease of the tissues supporting the tooth (16). Malocclusion in childhood is a risk factor for tooth wear in adulthood (17). Tooth wear can result in loss of enamel and exposed dentine hence causing sensitivity which sometimes requires a root canal (18). Irregular teeth have also been identified as a cause of gingival recession among children (19). Gum recession generally occurs due to periodontal trauma caused by rotated, crowded, or tipped teeth (20, 21). Temporomandibular disorders (TMD) are defined as a collection of disorders that affect the temporomandibular joints, facial nerves, and muscles of the jaw. If joints, bones, and muscles are unable to work in conjunction with one another for any reason, it can lead to TMD (22, 23). Malocclusions increase the risk of developing TMDs (24). Even though it can be treated (24), there are no criteria to determine which if any patient would recover (25). Children under 12 are in a critical stage of occlusal development, as mixed dentition gives way to early permanent dentition, with major changes in arch length, eruption, and inter-arch relationships. This phase is marked by active occlusal changes, and several malocclusal patterns observed during this period may be partially or fully self-corrected as permanent teeth erupt and occupational relationships are established (26, 27). Longitudinal studies have shown that some of the following irregularities in the occlusals may resolve naturally, but others persist and increase in the later stages of development, which explains the relevance of early detection at this stage (26, 27). Investigating malocclusion in children under 12 years old thus provides an opportunity to evaluate malocclusion when interceptive treatments are most efficient and clinically important to differentiate between temporary and possibly long-term occlusal defects. Unlike most preceding studies that use adolescents or a general age group, the study at hand directly focuses on this developmental period, thereby increasing its developmental significance and adding new knowledge to the evidence concerning the linkage between malocclusion and related occlusal situations in early dentofacial development.

The literature shows that previous studies have evaluated the role played by malocclusion in future occlusion problems (26–30). However, these studies were only focused on associating malocclusion with one problem such as oral dysfunction (31), headaches (32), and craniomandibular disorders (33). The present study aims to focus on a range of occlusion problems that can be encountered later in life as a result of malocclusion. Furthermore, this study would also include a comparative analysis of occlusion problems between mixed and permanent dentitions in children. These findings would help understand the multifaceted impact of malocclusion as they provide valuable evidence to develop specific treatment approaches and other preventative strategies. It would also guide the clinicians to prioritize oral health interventions to enhance overall patient outcomes.

## 2 Materials and methods

### 2.1 Study design

A cross-sectional study was conducted among pediatric patients who presented for routine dental check-ups at Taibah University Hospital.

### 2.2 Study period

The study was conducted during the recruitment period (August 2024 to April 2025). Individuals were enrolled in the study during routine outpatient oral health visits.

### 2.3 Sampling strategy

A convenient sampling method was used. All children who visited the dental clinic within the study period and met the eligibility requirements were invited to participate.

### 2.4 Eligibility criteria

Children involved in the study if they were under the age of 12 years, visit the clinic to have their teeth examined, and they must not have any prior orthodontic treatment history, either in the form of fixed or removable appliances. The participants were included irrespective of having malocclusion or not. Exclusion criteria included children with known congenital craniofacial anomalies, including cleft lip and/or palate, systemic conditions that impaired craniofacial growth and development, or insufficient cooperation to undergo the clinical examination.

### 2.5 Eligibility screening and consent

Eligibility screening was conducted by a trained dental examiner before the participant was included in the study. Parents or legal guardians were required to sign informed consent before their participation.

### 2.6 Sample size

A single-proportion estimation method was used to compute the minimum sample size. The predicted prevalence of malocclusion was 71%, determined in a longitudinal study conducted when primary dentition was replaced by early permanent dentition (27). The required sample size was 317 persons at the 95% confidence level with a precision of 5%. Since this cross-sectional clinical examination study did not involve a loss to follow-up, no dropout adjustment was implemented. The sample of 500 children thus had a satisfactory sample size, and consequently, sufficient accuracy in estimating prevalence and conducting exploratory multivariate analyses.

### 2.7 Ethical approval

The study was reviewed and approved by the Research Ethics Committee of the College of Dentistry, Taibah University, Ministry of Education. Ethical approval was granted under the reference number TUCD-REC (TUCDREC/141124). All procedures were conducted in accordance with the ethical standards of the institutional committee.

### 2.8 Clinical examination, diagnostic criteria

A standardized intraoral clinical assessment of each respondent was performed in a proper setting of light with the help of mouth mirrors and dental explorers. Tests were conducted by two or more qualified dentists in accordance with established, standardized evaluation procedures to ensure transparency and consistency in data collection. The evaluation of malocclusion was performed using a simplified clinical methodology based on Angle classification, whereby occlusion was classified as normal or malocclusion (Class I, Class II, or Class III) (34). Moreover, the clinically significant irregularities of the occlusality (crowding, spacing, excessive overjet, deep bite, open bite or crossbite) were documented. To be analytical, the malocclusion variable was dichotomized (present/absent). Children who looked like they had at least one abnormal occlusal feature were labeled as having malocclusion, and those who had no abnormal features were labeled as not having malocclusion. Such a dichotomous methodology corresponds with the methodologies applied in other prior population-based epidemiological studies of children (35, 36). The status of dentition was clinically assessed and classified as mixed dentition (carrying of both primary and permanent teeth) or permanent dentition (carrying of permanent teeth only). No early and late mixed dentition was made. The assessment of tooth wear was conducted using a clinical screening tool based on the Smith and Knight Tooth Wear Index (37). When wear was first detected in enamel or dentin in the clinical examination in at least one tooth, this was recorded. Tooth wear was measured as a dichotomous variable (yes/no), but no measure of the severity of wear. Traumatic dental injury (TDI) was determined using clinical outcomes and dental history that agrees with the Andreasen classification of dental trauma, encompassing enamel fractures and enamel–dentin fractures, and tooth displacement injuries (34, 38). TDI was registered as present or absent based on the identification of any past or current traumatic injury. Gingival recession was considered apical loss of the gingival margin to a position posterior to the cemento-enamel junction and was classified as either present or absent in case it was observed in any of the surfaces of the tooth. Calibrated dental practitioners performed all clinical examinations and were trained before data collection during joint clinical sessions and agreement on diagnostic criteria. Although no formal inter and intra examiner reliability testing was conducted, standardized diagnostic criteria and examination procedures were used to minimize variability in the assessment.

### 2.9 Statistical analysis

Data were analyzed using IBM SPSS Statistics Version 22 (IBM Corp., Armonk, NY, USA). Descriptive statistics were presented using frequencies and percentages. The differences between malocclusion and categorical oral health variables (tooth wear, traumatic dental injury, gingival recession, and dentition type) were compared with the help of the Pearson chi-square test. The exact test was used at Fisher, where the expected count of cases was below five. logistic regression analysis was done to investigate the factors related to occurrence of malocclusion. Malocclusion was treated as a binary dependent variable (present/absent), an approach commonly adopted in population-based pediatric studies to enable multivariable logistic regression analysis and comparison with existing literature (35, 36), and the independent variables were, tooth wear, traumatic dental injury, gingival recession, and dentition type. Dentition was categorized into one variable to avoid repetition. The chi-square goodness-of-fit test and Nagelkerke R^2^ were used to assess model fit. For multivariable analysis, dentition was modeled as a binary variable (permanent vs. not permanent), with the reference category including children in deciduous and mixed dentition Statistical significance was assessed at *p* < 0.05.

## 3 Results

Our study results include 249(49.8%) Males and 251(50.2%) Females. The majority of children were aged between 5 and 8 years, 269 (53.8%), and 231 (46.2%) were between the age from 9 to 11 years. 252 (50.4%) had permanent dentition, 252 (50.4%) of children had no tooth wear, 264 (52.8%) had traumatic dental injuries, and 258 (51.6%) had receding gum (Table 1).

**Table 1:** Demographic characteristics.

Demographic characteristics		*n* (%)
Age	5–8 Years	269 (53.8%)
	9–11 Years	231 (46.2%)
Gender	Male	249 (49.8%)
	Female	251 (50.2%)
Dentition Type	Permanent	252 (50.4%)
	Mixed	248 (49.6%)
Tooth wear	Yes	248 (49.6%)
	No	252 (50.4%)
Traumatic dental injury	Yes	264 (52.8%)
	No	236 (47.2%)
Receding gums	Yes	258 (51.6%)
	No	242 (48.4%)

The association between malocclusion and related oral health issues among the 500 participants was analyzed using the chi-square test (Table 2). Participants with malocclusion showed more tooth wear (54.2%) than those without malocclusion (45.4%). Similarly, 57.6% of people with malocclusion had permanent dentition, compared with 42.4% without malocclusion (*χ*^2^ = 5.670, *p* = 0.017). However, no important relationship was found between malocclusion, mixed dentition and traumatic dental injury.

**Table 2:** Association between malocclusion and other oral health variables (*n* = 500).

Variables		Malocclusion		*χ* ^2^	*p*-value
		Yes	No		
		*n* (%)	*n* (%)		
Tooth wear	Yes	141 (54.2%)	109 (45.4%)	3.878	0.049
	No	119 (45.8%)	131 (54.6%)		
Traumatic dental_injury	Yes	128 (49.2%)	112 (46.7%)	0.329	0.566
	No	132 (50.8%)	128 (53.3%)		
Dentition permanent	Yes	136 (52.3%)	100 (41.7%)	5.670	0.017
	No	124 (47.7%)	140 (58.3%)		
Dentition mixed	Yes	128 (49.2%)	124 (51.7%)	0.296	0.586
	No	132 (50.8%)	116 (48.3%)		

The multivariate regression findings are presented in Table 3 and used to investigate factors associated with malocclusion in the study population. Tooth wear, traumatic dental injury (TDI), and dentition type were included as independent variables in the model. After adjusting for all variables, tooth wear was significantly associated with malocclusion (adjusted OR = 0.69; 95% CI: 0.49–0.99; *p* = 0.043). Children with tooth wear had 31% lower odds of presenting with malocclusion than those without tooth wear. Malocclusion was also strongly associated with tooth dentition type. Odds of malocclusion were significantly lower in children with permanent dentition than in those without permanent dentition (i.e., children with mixed or deciduous dentition) (adjusted OR = 0.64; 95% CI: 0.45–0.91; *p* = 0.014), indicating a 36% reduction in the likelihood of malocclusion among children with permanent dentition. In contrast, the adjusted model showed no significant association between traumatic dental injury (TDI) and malocclusion (adjusted OR = 0.91; 95% CI: 0.64–1.29; *p* = 0.593), suggesting that a history of dental trauma was not significantly associated with malocclusion in this population. The overall model was statistically significant (Omnibus *χ*^2^ = 10.53, df = 3, *p* = 0.015), indicating that the included variables collectively explained variation in malocclusion status. However, the model exhibited low explanatory power, as reflected by the Cox and Snell *R*^2^ (0.021) and Nagelkerke *R*^2^ (0.028). The overall classification accuracy was 57.4% also indicates limited predictive ability, highlighting that malocclusion is influenced by various developmental, functional, behavioral, and contextual factors not incorporated into the current analysis.

**Table 3:** Multivariable logistic regression analysis for factors associated with malocclusion.

Variable	Category	*B*	S.E.	Wald *χ*^2^	*p*-value	Adjusted OR	95% CI for OR
Tooth wear (TW)	Yes	−0.367	0.181	4.098	0.043*	0.69	0.49–0.99
	No	(Reference)					
Traumatic dental injury (TDI)	Yes	−0.097	0.181	0.286	0.593	0.91	0.64–1.29
	No	(Reference)					
Dentition type	Permanent	−0.448	0.182	6.073	0.014*	0.64	0.45–0.91
	Not permanent^†^	(Reference)					
Constant	—	0.306	0.198	2.396	0.122	1.36	—

**Model statistics:.**

• Omnibus Test of Model Coefficients: *χ*^2^ = 10.53, *df* = 3, *p* = 0.015.

• −2 Log Likelihood: 681.82.

• Cox & Snell *R*^2^ = 0.021.

• Nagelkerke *R*^2^ = 0.028.

• Overall Classification Accuracy = 57.4%.

*Significant *p*-values.

† Reference category (“not permanent”) includes children with deciduous and mixed dentition.

## 4 Discussion

In this cross-sectional study, the relationship between malocclusion and oral health variables in children under 12 years was examined, as this is a developmental period between the late mixed dentition and early permanent dentition stages. In the bivariate analyses, malocclusion was significantly correlated with tooth wear and permanent dentition status, but not with traumatic dental injury (TDI) and mixed dentition status. Tooth wear (adjusted OR = 0.69) and permanent dentition (adjusted OR = 0.64) were also significant in their multivariate relationship with malocclusion and showed a negative association, whereas TDI was not significant. However, the model accounted for a low percentage of variance (Nagelkerke *R*^2^ = 0.028), indicating that it could not be used to predict outcomes effectively and confirming that malocclusion is dependent on many developmental, functional, behavioral, and contextual factors (31, 38). This also shows that children with permanent dentition had a lower probability of being maladjusted than the reference category (not permanent, i.e., mixed or deciduous dentition). This trend can be explained within the framework of occlusal development. Mixed dentition is a biologically dynamic phase characterized by periodic irregularities in eruption, exfoliation, and space changes, which can obscure occlusal changes in cross-sectional snapshots (27, 30). Some of the temporary characteristics stabilize as the occlusion matures and the permanent dentition is determined, it is less likely that a child will fit into a wide-ranging definition of malocclusion as anything that exists. This developmental explanation is consistent with the epidemiologic literature indicating variation in malocclusion prevalence and occlusal trait patterns across age and dentition stages in populations (39–41). This also supports the clinical rationale for screening at school age, when malocclusal characteristics are often noted and interceptive treatment options are available (42, 43). Notably, since the dentition stage is a marker of the timing of development, the observed negative correlation here should not be seen as a protective effect. rather, this probably represents the volatility of the occlusal traits in the transition period and the inability of cross-sectional measurement to capture a time-dependent condition (27).

Mixed dentition status was not significantly correlated with malocclusion in the analyses. Heterogeneity within the mixed dentition group is one possibility, early mixed dentition can be characterized by temporary crowding, incisal transitions, and space redistribution, while late mixed dentition may show emerging stability or other manifestations of trait expression. Trait-level signals can be diluted when early and late mixed dentition are not distinguished, consistent with broader orthodontic literature indicating that eruption stage affects trait visibility and treatment need (43).

Tooth wear was strongly correlated with malocclusion in the bivariate analysis and remained significant in the regression, with an adjusted odds ratio below 1, indicating a lower likelihood of malocclusion among children with tooth wear. Such directionality is the opposite extreme to a clinical belief that some types of malocclusion (e.g., deep bite, crossbite with functional shifts, or interferences) might be involved in wear due to altered contacts and guidance. associations between malocclusion and wear depend strongly on how malocclusion is operationalized (binary vs. trait-specific), whether wear is physiological or pathological, and whether wear severity is graded (17, 18). Tooth wear in the current study was dichotomized (present on at least one tooth), but it may reflect a combination of physiological wear (which should occur in primary teeth) and pathological wear (which may be associated with erosion or parafunction). Thus, the above inverse relationship must be viewed with caution and does not mean that tooth wear decreases the risk of malocclusion. Mechanistically, tooth wear patterns of the tooth in children could be indicative of more extensive functional and behavioral exposures like dietary acids, parafunctional habits, or patterns of occlusal functions, which might interact with characteristics of malocclusion in more complex ways (17, 18, 44). Validated pediatric wear indices and severity grading should be employed in future studies, and separation of physiological wear from erosive or attritional patterns of wear is needed to determine whether particular occlusal features are related to clinically meaningful patterns of wear (18).

Malocclusion was not associated with TDI. This result is contrary to the literature, which states that particular malocclusion features, especially overjet augmentation and insufficient lip coverage, are linked to a greater risk of TDI (45–47). It may be a methodological divergence, researchers that find significant connections with trauma tend to focus on single traits that are related to trauma (e.g., large overjet) instead of applying a broad dichotomous outcome that combines heterogeneous malocclusion characteristics (14, 45). Additionally, behavior, environment, physical activity, supervision, and socioeconomic context affect the TDI risk but were not measured here and could dominate occlusal effects in typical clinical populations (38). These points justify the suggestion that trait-specific malocclusion indicators, in particular, overjet and lip competence, should be considered in future studies in the modeling with behavioral and contextual covariates of TDI risk (46, 47).

Gingival recession was more common in this sample (51.6%). Because recession was not included in the multivariate model, no conclusion can be drawn about its adjusted relationship with malocclusion. Although reported associations are also evident in other environments, plaque control, inflammation, brushing trauma, and anatomy are considered to have a strong impact on periodontal outcomes, and only some of the malocclusion characteristics (e.g., crowding/irregularity) might have a weak relationship in particular circumstances (15). Given the unexpected high prevalence, future efforts should confirm diagnostic thresholds, retain the severity grading, and incorporate periodontal risk factors to prevent overattributing recession patterns to occlusal status alone (15).

These findings have several constraints that should be considered when interpreting the results. the study design is cross-sectional and cannot establish temporal relationships or causality. Malocclusion was measured as a binary outcome (present/absent). This is a common approach in pediatric epidemiologic research for estimating prevalence and building models, especially when power is limited in a trait-level distribution, but it comes at the cost of reduced clinical specificity and may obscure trait-specific relationships (35, 36). Tooth wear was measured dichotomously without severity grading or a physiological/pathological distinction, which limited its interpretation (17, 18). No formal inter- and intra-examiner reliability testing has been conducted, which could have introduced measurement variation despite the use of standardized criteria. Lastly, the multivariate model was poor (Nagelkerke *R*^2^ = 0.028), which is consistent with the multifactorial etiology of malocclusion and the probable role of unmeasured factors, including oral habits, mouth breathing, early childhood tooth loss, and socioeconomic background (31, 38, 48).

These findings are clinically relevant as they suggest that early occlusal evaluation of the mixed dentition transition to permanent dentition is needed, the observation of malocclusions in the mixed dentition might be a temporary developmental phenomenon and does not indicate a chronic malocclusion that requires urgent treatment. Longitudinal designs would be required to distinguish between transient and persistent malocclusion trends across stages of eruption and to enhance explanatory models by including functional, behavioral, and socioeconomic factors (27, 38).

## 5 Conclusion

In Conclusion, the results suggest that malocclusion in this pediatric group was negatively correlated with tooth wear and permanent dentition status, whereas traumatic dental injury and mixed dentition were not significantly correlated. The low explanatory power of the regression model confirms the multifactorial and developmentally dynamic nature of malocclusion and discourages causation. It may be necessary to consider occlusal irregularities at the mixed-to-permanent dentition transition as a transient developmental phenomenon rather than a lasting malocclusion. These results support the value of early assessment of occlusivity and longitudinal evaluation as opposed to untimely orthodontic treatment.

## Data Availability

The raw data supporting the conclusions of this article will be made available by the authors, without undue reservation.

## References

[1] Baskaradoss JK Bhagavatula P. Measurement and distribution of malocclusion, trauma, and congenital anomalies. Dent Pract Community. (2021) 2:208–17. 10.1016/B978-0-323-55484-8.00018-6

[2] Stomatologic SI. Worldwide prevalence of malocclusion in the different stages of dentition: a systematic review and meta-analysis. Eur J Paediatr Dent. (2020) 21:115. 10.23804/ejpd.2020.21.02.05 32567942

[3] World Health Organization. Health Through Oral Health: Guidelines for Planning and Monitoring for Oral Health Care. Geneva: WHO (1989).

[4] Gudipaneni RK Aldahmeshi RF Patil SR Alam MK. The prevalence of malocclusion and the need for orthodontic treatment among adolescents in the northern border region of Saudi Arabia: an epidemiological study. BMC Oral Health. (2018) 18:16. 10.1186/s12903-018-0476-8 29390986 PMC5796577

[5] Chaturvedi TP Mishra R Sharma VK Aparna AV Nagvanshi S. Genes to jaws: a systematic review uncovering the role of genetics in malocclusion. Gene Rep. (2025) 42:102388. 10.1016/j.genrep.2025.102388

[6] Sadoun C Templier L Alloul L Rossi C Diaz Renovales I Nieto Sanchez I Effects of non-nutritive sucking habits on malocclusions: a systematic review. J Clin Pediatr Dent. (2024) 48:4–18. 10.22514/jocpd.2024.029 38548628

[7] Moimaz SAS Garbin AJI Lima AMC Lolli LF Saliba O Garbin CAAS. Longitudinal study of habits leading to malocclusion development in childhood. BMC Oral Health. (2014) 14:96. 10.1186/1472-6831-14-96 25091288 PMC4126276

[8] Ali F Soni S Kaur G Bagga MK. Oral habits in relation to malocclusions: a review. Int J Health Sci. (2021) 15:230–8. 10.53730/ijhs.v5nS2.5660

[9] Littlefield WH. Thumb-sucking and its relationship to malocclusion in children. Am J Orthod. (1952) 38:293–300. 10.1016/0002-9416(52)90122-X

[10] Chen H Lin L Chen J Huang F. Prevalence of malocclusion traits in primary dentition: a systematic review. Healthcare. (2024) 12:1321. 10.3390/healthcare12131321 38998856 PMC11241413

[11] Garbin AJ Perin PC Garbin CA Lolli LF. Malocclusion prevalence and comparison between the angle classification and the dental aesthetic index in scholars in São Paulo state, Brazil. Dent Press J Orthod. (2010) 15:94–102. 10.1590/S2176-94512010000400014

[12] Majid ZS Abidia RF. Effects of malocclusion on oral health-related quality of life: a critical review. Eur Sci J. (2015) 11:1–8.

[13] English JD Buschang PH Throckmorton GS. Does malocclusion affect masticatory performance? Angle Orthod. (2002) 72:21–7. 10.1043/0003-3219(2002)072<0021:DMAMP>2.0.CO;2 11843269

[14] Petti S. Over two hundred million injuries to anterior teeth attributable to large overjet: a meta-analysis. Dent Traumatol. (2015) 31:1–8. 10.1111/edt.12126 25263806

[15] Alsulaiman AA Kaye E Jones J Cabral H Leone C Will L Incisor malalignment and the risk of periodontal disease progression. Am J Orthod Dentofacial Orthop. (2018) 153:512–22. 10.1016/j.ajodo.2017.08.015 29602343

[16] Könönen E Gursoy M Gursoy UK. Periodontitis: a multifaceted disease of tooth-supporting tissues. J Clin Med. (2019) 8:1135. 10.3390/jcm8081135 31370168 PMC6723779

[17] Cunha-Cruz J Pashova H Packard JD Zhou L Hilton TJ. Tooth wear: prevalence and associated factors in general practice patients. Community Dent Oral Epidemiol. (2010) 38:228–34. 10.1111/j.1600-0528.2010.00537.x 20370807 PMC3116086

[18] Addy M. Tooth brushing, tooth wear and dentine hypersensitivity—are they associated? Int Dent J. (2005) 55:261–7. 10.1111/j.1875-595X.2005.tb00063.x 16167604

[19] Nwhator SO. Gingival recession in a child patient; easily missed etiologies: case report. Ann Med Health Sci Res. (2014) 4:18–21. 10.4103/2141-9248.131698 25031899 PMC4083724

[20] Kumar J Kumar S Doraiswamy J Arun M. Association between gingival recession and malpositioned teeth: a retrospective cohort study. Int J Dent Oral Sci. (2019) 2:1–6. 10.19070/2377-8075-SI02-080010

[21] Marschner F Lechte C Kanzow P Hraský V Pfister W. Systematic review and meta-analysis on prevalence and risk factors for gingival recession. J Dent. (2025) 155:105645. 10.1016/j.jdent.2025.105645 39988303

[22] Ohrbach R Sharma S. Temporomandibular disorders: definition and etiology. Semin Orthod. (2024) 30:237–42. 10.1053/j.sodo.2023.12.011

[23] Hatfield E. An overview of diagnosis and management of temporomandibular disorders. J Calif Dent Assoc. (2024) 52:2400419. 10.1080/19424396.2024.2400419

[24] Abrahamsson C Henrikson T Nilner M Sunzel B Bondemark L Ekberg E. TMD before and after correction of dentofacial deformities by orthodontic and orthognathic treatment. Int J Oral Maxillofac Surg. (2013) 42:752–8. 10.1016/j.ijom.2012.10.016 23159168

[25] Al-Moraissi EA Wolford LM Perez D Laskin DM Ellis E III. Does orthognathic surgery cause or cure temporomandibular disorders? A systematic review and meta-analysis. J Oral Maxillofac Surg. (2017) 75:1835–47. 10.1016/j.joms.2017.03.029 28419845

[26] Różańska-Perlińska D Jaszczur-Nowicki J Kruczkowski D Bukowska JM. Dental malocclusion in mixed dentition children and its relation to podal system and gait parameters. Int J Environ Res Public Health. (2023) 20:2716. 10.3390/ijerph20032716 36768082 PMC9916284

[27] Dimberg L Lennartsson B Arnrup K Bondemark L. Prevalence and change of malocclusions from primary to early permanent dentition: a longitudinal study. Angle Orthod. (2015) 85:728–34. 10.2319/080414-542.1 25867255 PMC8610411

[28] Carlsson GE Egermark I Magnusson T. Predictors of bruxism, other oral parafunctions, and tooth wear over a 20-year follow-up period. J Orofac Pain. (2003) 17:50–7. 10.1016/S0889-5406(03)00245-2 12756931

[29] Myllymäki E Heikinheimo K Suominen A Evälahti M Michelotti A Svedström-Oristo AL Longitudinal trends in temporomandibular joint disorder symptoms, the impact of malocclusion and orthodontic treatment. J Oral Rehabil. (2023) 50:739–45. 10.1111/joor.13471 37102504

[30] Dos Santos CCO da Rosa Moreira Bastos RT Bellini-Pereira SA Garib D Normando D. Spontaneous changes in mandibular incisor crowding from mixed to permanent dentition: a systematic review. Prog Orthod. (2023) 24:15. 10.1186/s40510-023-00466-3 37150772 PMC10164666

[31] D’Onofrio L. Oral dysfunction as a cause of malocclusion. Orthod Craniofac Res. (2019) 22:43–8. 10.1111/ocr.12277 31074141 PMC6851783

[32] Lambourne C Lampasso J Buchanan WC Jr Dunford R McCall W. Malocclusion as a risk factor in the aetiology of headaches in children and adolescents. Am J Orthod Dentofacial Orthop. (2007) 132:754–61. 10.1016/j.ajodo.2006.03.033 18068593

[33] Egermark-Eriksson I Carlsson GE Magnusson T Thilander B. A longitudinal study on malocclusion and signs and symptoms of craniomandibular disorders in children and adolescents. Eur J Orthod. (1990) 12:399–407. 10.1093/ejo/12.4.399 2086260

[34] Angle EH. Classification of malocclusion. Dent Cosmos. (1899) 41:248–64.

[35] Assis WC Pereira JS Silva YS Brito FR Nunes LA Ribeiro IJS Factors associated with malocclusion in preschool children in a Brazilian small town. Pesqui Bras Odontopediatr Clin Integr. (2020) 20:e5351. 10.1590/pboci.2020.069

[36] Carvalho AA Almeida TF Cabral MBBS Cangussu MCT. Investigation of malocclusion and associated factors in preschoolers: a cross-sectional questionnaire study. Epidemiologia. (2024) 5:275–88. 10.3390/epidemiologia5020019 38920754 PMC11202454

[37] López-Frías FJ Castellanos-Cosano L Martín-González J Llamas-Carreras JM Segura-Egea JJ. Clinical measurement of tooth wear: tooth wear indices. J Clin Exp Dent. (2012) 4:e48–53. 10.4317/jced.50592 24558525 PMC3908810

[38] Tefera AT Bekele BG Derese K Andualem G. Prevalence of occlusal features and their relation to sociodemographic variables in Northwest Ethiopia. Clin Cosmet Investig Dent. (2021) 13:459–68. 10.2147/CCIDE.S332552 34785955 PMC8590399

[39] Xu J Li X Liu X Li S Lu Y. Prevalence and influencing factors of mixed dentition malocclusion in children in Jinzhou, China. Oral Health Prev Dent. (2023) 21:1–8. 10.3290/j.ohpd.b4100913 PMC11619866 37195332

[40] Yin J Zhang H Zeng X Yu J Wang H Jiang Y Prevalence and influencing factors of malocclusion in adolescents in Shanghai, China. BMC Oral Health. (2023) 23:590. 10.1186/s12903-023-03187-5 37620836 PMC10464309

[41] Lin L Chen W Zhong D Cai X Chen J Huang F. Prevalence and associated factors of malocclusion among preschool children in Huizhou. China. Healthcare. (2023) 11:1050. 10.3390/healthcare11071050 37046977 PMC10094330

[42] Alwadei SH Hattan AA Faqihi K Alhawiatan A Alwadei F Alwadei A. Prevalence of malocclusion and orthodontic treatment needs among Saudi primary school male children aged 6–12 years. J Int Oral Health. (2023) 15:106–12. 10.4103/jioh.jioh_159_22

[43] Madiraju GS Alnabi S Almarzooq A. Orthodontic treatment need and occlusal traits in early mixed dentition among Saudi children. Eur Oral Res. (2021) 55:110–5. 10.26650/eor.2021836877 34746781 PMC8547751

[44] Traebert E Zanini FA Nunes RD Traebert J. Nutritional and non-nutritional habits and occurrence of malocclusions in mixed dentition. An Acad Bras Cienc. (2020) 92:e20190833. 10.1590/0001-3765202020190833 32321028

[45] Arraj GP Rossi-Fedele G Doğramacı EJ. Association of overjet size and traumatic dental injuries: a systematic review and meta-analysis. Dent Traumatol. (2019) 35:217–32. 10.1111/edt.12481 31062510

[46] Cobourne MT DiBiase AT Seehra J Papageorgiou SN. Should we recommend early overjet reduction to prevent dental trauma? Br Dent J. (2022) 233:387–90. 10.1038/s41415-022-4916-0 36085463 PMC9463065

[47] Schatz JP Ostini E Hakeberg M Kiliaridis S. Large overjet as a risk factor of traumatic dental injuries: a prospective longitudinal study. Prog Orthod. (2020) 21:41. 10.1186/s40510-020-00341-5 33164157 PMC7649183

[48] Belitz GS Furlan LJ Knorst JK Berwig LC Ardenghi TM Ferrazzo VA Association between malocclusion in mixed dentition with breastfeeding and nonnutritive sucking habits. Angle Orthod. (2022) 92:669–76. 10.2319/111821-848.1 35759270 PMC9374351

